# Mechanism and Application of Biochar as an Electron Shuttle in the Remediation of Soil Pollutants

**DOI:** 10.3390/toxics14080708

**Published:** 2026-08-11

**Authors:** Lingling Feng, Ke Tan, Can Tang, Huan Zhao, Jiawen Zhu, Qingman Zhang, Jiayu Yan, Mengdi Xie

**Affiliations:** 1 College of Ecology and Environment, Chengdu University of Technology, Chengdu 610059, China; fenglingling@stu.cdut.edu.cn (L.F.); 2025051429@stu.cdut.edu.cn (K.T.); tangcan@stu.cdut.edu.cn (C.T.); zhaohuan@stu.cdut.edu.cn (H.Z.); 15208153034@163.com (J.Z.); 15145310913@163.com (Q.Z.); 17311298791@163.com (J.Y.); 2 Key Laboratory of Water and Soil Pollution Collaborative Control and Joint Restoration, Chengdu 610059, China

**Keywords:** biochar, electron shuttle, soil remediation, heavy metals, organic pollutants, immobilization

## Abstract

Biochar has attracted increasing attention in soil remediation due to its porous structure, abundant surface functional groups, and environmental compatibility. Beyond its conventional roles as an adsorbent and soil amendment, biochar has increasingly been recognized as an electron shuttle that mediates electron transfer between electron donors and acceptors, thereby promoting redox reactions involved in pollutant transformation, immobilization, and toxicity mitigation. However, the structural basis, influencing factors, and underlying electron-transfer mechanisms of biochar-mediated processes remain insufficiently integrated. This review summarizes biochar-mediated electron transfer in soil remediation. Three pathways are discussed: direct electron transfer through conductive carbon matrices, indirect electron transfer mediated by redox-active surface functional groups, and composite interfacial electron transfer involving microorganisms, metal oxides, and nanomaterials. By introducing the synergistic effects between biochar and microorganisms, metal oxides, and nanomaterials, the functions of biochar in immobilizing heavy metals and degrading organic pollutants are emphasized. Biochar’s electron-shuttling ability is closely related to aromatic carbon structures, redox-active functional groups, and persistent free radicals, while key factors affecting electron-shuttling performance, including feedstock properties, pyrolysis temperature, pH, coexisting ions, pollutant concentration, and modification strategies, are also discussed. This review offers guidance for the design of biochar-based soil remediation strategies.

## 1. Introduction

Soil pollution caused by heavy metals and organic contaminants has become a global environmental concern, posing risks to soil quality, ecosystem stability, and human health [1]. Biochar, a carbonaceous material derived from biomass pyrolysis, has been widely applied in soil remediation due to its low cost, porous structure, large specific surface area, and diverse surface functional groups [2,3]. Traditionally, biochar has been regarded mainly as an adsorbent and soil amendment that reduces pollutant mobility and bioavailability through adsorption, ion exchange, surface complexation, precipitation, and electrostatic interactions [4]. These mechanisms explain the immobilization of many pollutants, particularly heavy metals, but do not fully account for pollutant transformation processes involving redox reactions. Because redox reactions are fundamentally driven by electron transfer, increasing attention has been paid to the role of biochar in mediating electron transfer processes in soil systems. In this context, biochar is no longer considered merely a passive remediation material; rather, its redox-active functional groups and conductive carbon matrix enable it to participate in electron-transfer processes associated with pollutant transformation and speciation regulation.

Electron shuttling is a key form of biochar-mediated electron transfer, in which an electron shuttle can reversibly accept electrons from electron donors and transfer them to electron acceptors, thereby accelerating redox reactions that would otherwise be limited by the requirement for direct contact among reactants. In this review, electron transfer refers to the movement of electrons between electron donors and acceptors during pollutant transformation processes. Electron transport describes the migration of electrons through conductive pathways or interfacial networks. An electron mediator is a broader term describing a component that facilitates electron transfer between reaction partners, with electron shuttles representing mediators possessing reversible electron storage and release capability. Accordingly, biochar can function as an electron shuttle or electron mediator depending on its structural characteristics, redox-active sites, and interactions with surrounding environmental components. The concept of electron shuttling originated from early studies on microbial electron transfer. Potter (1911) first demonstrated that microorganisms could generate electric current during organic matter decomposition, laying the foundation for understanding extracellular electron transfer [5].

Subsequent work showed that humic substances can act as reversible electron carriers, transferring electrons between microbial cells and insoluble electron acceptors such as Fe(III) oxides [6]. More recently, Kappler et al. [7] demonstrated that biochar can mediate electron transfer between microorganisms and minerals via redox cycling of surface quinone-like groups and direct electron transfer through conductive carbon domains within biochar. Sun et al. [8] further revealed that the graphene-like carbon structures of biochar support long-range electron transfer, indicating its active role in facilitating pollutant transformation in soil.

Despite growing interest in biochar as an electron shuttle, current studies remain distributed across diverse environmental systems without a unified mechanistic framework. Previous reviews have mainly focused on the adsorption capacity, physicochemical properties, modification strategies, and general remediation performance of biochar in contaminated soils, particularly for heavy metal immobilization. Although these studies have significantly advanced the understanding of biochar applications, the intrinsic relationship between biochar structural characteristics and electron-shuttling behavior remains insufficiently understood. In particular, the roles of aromatic carbon structures, graphitic domains, redox-active functional groups, heteroatom-containing defects, and persistent free radicals in regulating electron storage, transport, and exchange processes remain insufficiently integrated.

Furthermore, biochar-mediated electron transfer has been investigated in various environmental systems, including microbial remediation, heavy metal transformation, organic pollutant degradation, and biochar-based composite materials. However, these studies generally focus on individual systems, resulting in fragmented interpretations of biochar-mediated electron-transfer mechanisms. A comprehensive framework linking biochar properties, electron-transfer pathways, and pollutant transformation processes is still lacking. To clarify the novelty and scope of this review, recent reviews related to biochar-based environmental remediation and electron transfer behavior were compared according to their research focus and mechanistic perspectives (Table 1).

**Figure 1:**
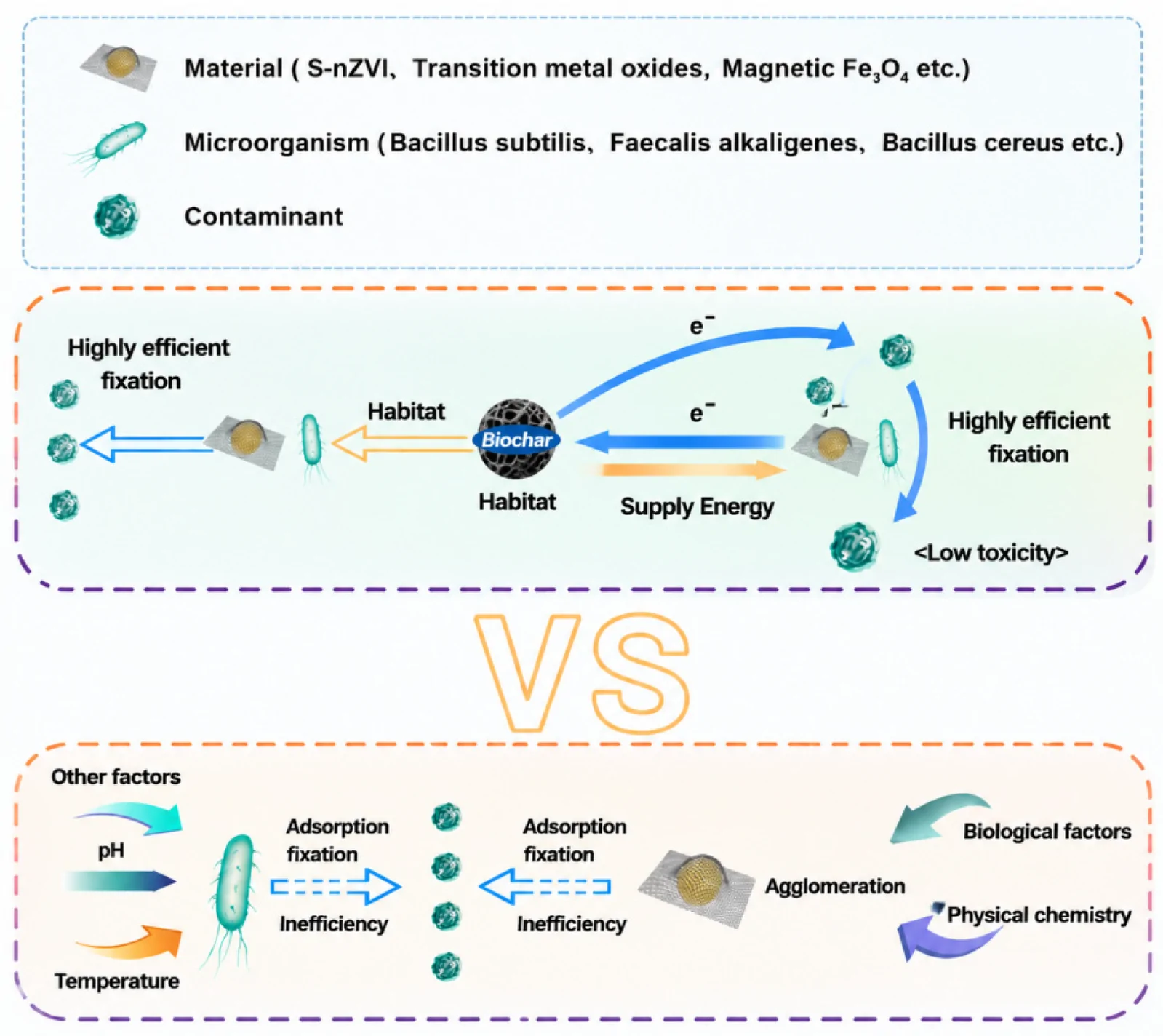
Conceptual comparison of pollutant transformation processes in the presence and absence of biochar, highlighting the role of biochar in enhancing interfacial electron transfer and remediation efficiency.

Therefore, this review systematically summarizes the electron-shuttling mechanisms of biochar in soil remediation by establishing a structure-property-function perspective. The structural factors controlling biochar electron-shuttling capability, including conductive carbon domains, redox-active functional groups, heteroatom-containing structures, and persistent free radicals, are first discussed. Subsequently, the interactions between biochar and environmental components, such as microorganisms, metal oxides, and nanomaterials, are analyzed to elucidate their roles in regulating interfacial electron transfer. The contributions of electron-transfer processes to heavy-metal immobilization and organic-pollutant transformation are further summarized. Finally, the effects of environmental conditions on biochar electron-shuttling performance are evaluated. Through these analyses, this review aims to establish a framework linking biochar physicochemical characteristics, electron-transfer pathways, and pollutant-transformation behaviors, as conceptually illustrated in Figure 1 through a comparison of remediation processes in the presence and absence of biochar.

### Literature Selection and Search Strategy

A literature search was conducted to collect publications related to biochar-mediated electron transfer and soil pollutant remediation. Relevant studies were identified from the Web of Science Core Collection, Scopus, and Google Scholar databases using combinations of keywords, including “biochar”, “electron transfer”, “electron shuttle”, “redox activity”, “soil remediation”, “heavy metals”, “organic pollutants”, “microorganisms”, and “biochar composites”. Studies published mainly between 2000 and 2026 were considered.

**Figure 2:**
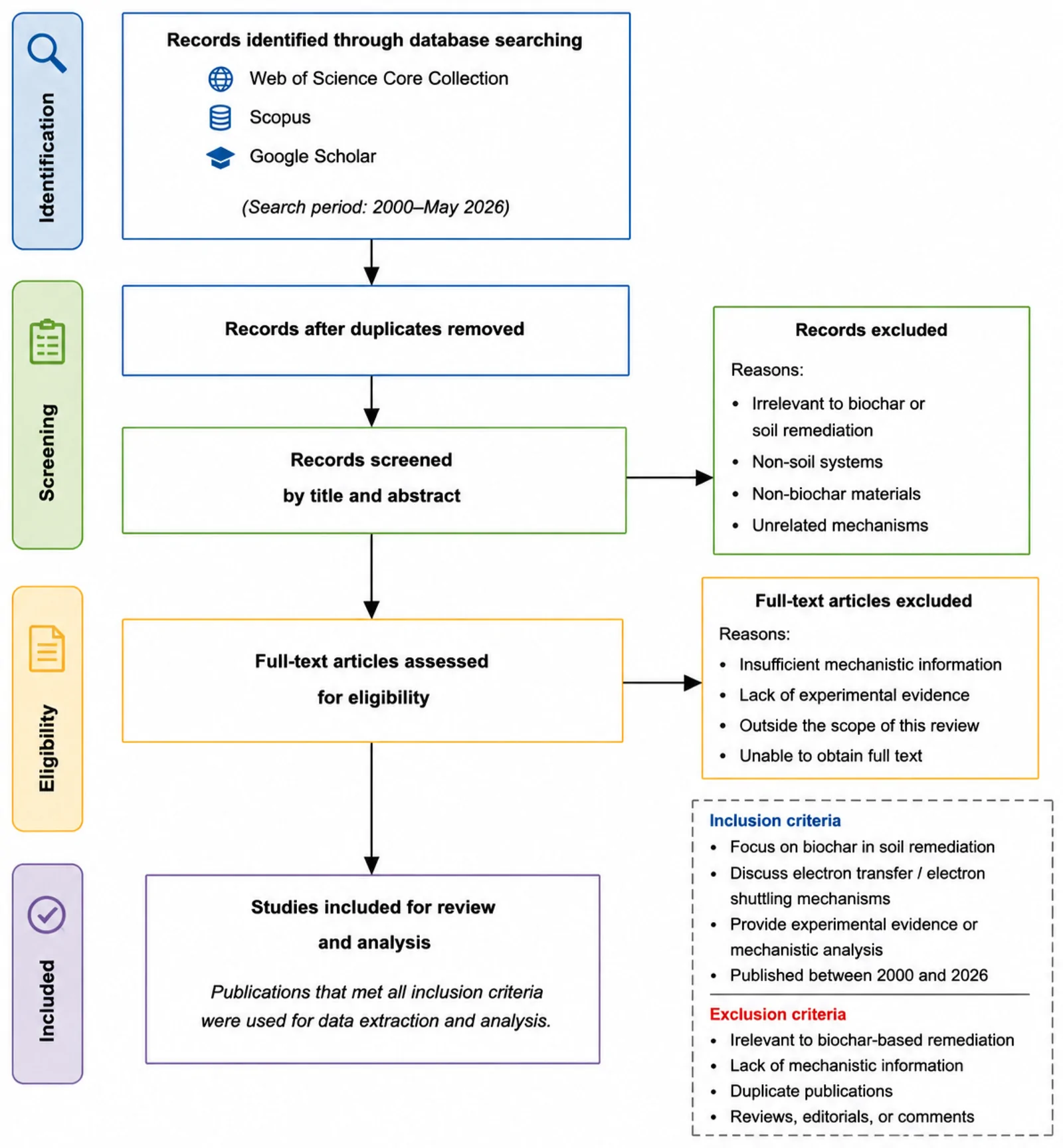
PRISMA flow diagram illustrating the literature search and selection process used in this review. (This figure was refined with the assistance of ChatGPT (OpenAI GPT-5.5-mini)).

The selected publications were screened based on their relevance to the objectives of this review, including (i) structural characteristics associated with biochar electron transfer behavior, (ii) experimental approaches for evaluating electron-shuttling capability, (iii) interactions between biochar and microorganisms, minerals, or nanomaterials, and (iv) applications of biochar-mediated electron transfer in pollutant remediation. Studies focusing only on conventional adsorption processes without discussion of electron-transfer-related mechanisms were not considered as core references. Additional references were identified from the citation lists of selected articles to include representative mechanisms and recent developments, and the overall literature identification, screening, eligibility assessment, and inclusion process is summarized in Figure 2.

## 2. Biochar-Based Interfacial Systems Enabling Electron Exchange

Biochar-mediated electron exchange is not determined solely by the intrinsic properties of biochar, but also depends on the interfacial interactions between biochar and surrounding components, including microorganisms, minerals, and functional nanomaterials. Therefore, this section focuses on the structural components and interfacial configurations that enable electron exchange in biochar-based systems.

### 2.1. Biochar System

Biochar itself possesses structural and redox-active features that allow it to function as an electron shuttle in environmental remediation systems. Previous studies have shown that nitrogen vacancy defects and graphitic nitrogen structures on the surface of biochar are important for electron-transfer processes, because these structures can provide active sites for electron acceptance, storage, and release [12]. In addition, the redox properties of biochar enable it to reversibly store and donate electrons, allowing biochar to mediate electron exchange between electron donors and electron acceptors.

Aromatic carbon structures are another important basis for the electron-shuttling behavior of biochar. Biochar is rich in aromatic carbon domains, and the distribution of π electrons within these structures allows biochar to interact with pollutants containing π-electron systems [13]. For example, methylene blue and phenolic pollutants can be adsorbed onto the surface of biochar through π–π interactions between their aromatic rings and the aromatic structures of biochar. Under neutral conditions, oxygen-containing functional groups on the biochar surface, such as -COOH and -OH, may act as π-electron donors and interact with phenolic compounds as π-electron acceptors, thereby strengthening pollutant adsorption and improving interfacial contact for subsequent electron-transfer processes.

Although adsorption is an important process, the role of biochar is not limited to pollutant enrichment on its surface. The aromatic carbon matrix, N-containing structures, and oxygen-containing functional groups can jointly create favorable conditions for interfacial electron transfer at the biochar–pollutant interface. Therefore, biochar can participate in redox-related environmental processes, such as nitrate reduction during denitrification [14] and the direct or mediated reduction of hexavalent chromium [15]. These processes indicate that biochar can act not only as an adsorbent but also as an active electron transfer medium in environmental biogeochemical reactions.

### 2.2. Biochar and Microbial Organisms

Microorganisms have shown great potential in the remediation of heavy metal and organic-pollutant contamination. Bacteria, fungi, and algae can accumulate heavy metals through surface adsorption, ion exchange, intracellular accumulation, and extracellular precipitation [16]. However, free microorganisms are often sensitive to environmental conditions such as temperature, pH, nutrient availability, and pollutant toxicity, which may lead to unstable remediation efficiency under practical soil conditions. Biochar can partly overcome these limitations because of its porous structure, high specific surface area, surface functional groups, and electrical conductivity. These properties enable biochar to serve not only as a carrier for microbial immobilization but also as an electron shuttle or mediator that facilitates interfacial electron transfer in biochar–microorganism remediation systems [17].

(1) Carrier function of biochar and immobilization technology

As a microbial carrier, biochar can provide attachment sites and protective microhabitats for functional microorganisms, thereby improving their environmental adaptability and survival. Its porous structure can physically protect microbial cells from pollutant stress, while its surface chemical properties facilitate microbial adhesion and biofilm formation, which promotes close contact between microorganisms and biochar interfaces. Previous studies on biochar-immobilized enzyme technology have confirmed that the surface properties of biochar are favorable for biological attachment and catalytic stability through enhanced microbial-material interactions [18]. For example, rice husk biochar was used to immobilize Alcaligenes faecalis JH1, and the immobilized system maintained stable phenol degradation performance in a 300 mg/L phenol solution within 12 h, while also showing good recyclability [19]. Similarly, a biochar-based Bacillus subtilis inoculant improved bacterial protection and functional stability, increasing plant biomass and reducing disease incidence compared with the non-immobilized treatment [20]. These results indicate that biochar immobilization can enhance microbial persistence and maintain pollutant degradation efficiency over repeated or long-term applications. In addition, biochar inoculated with functional bacteria can promote plant growth more effectively than biochar alone, suggesting that biochar-microorganism systems may provide both remediation and agronomic benefits [21].

(2) Electron transfer mechanism and degradation of organic pollutants

In addition to its carrier function, biochar can participate in microbial pollutant degradation by mediating electron transfer. The conductive carbon matrix of biochar provides a pathway for electron movement, while surface functional groups such as carboxyl and carbonyl groups may interact with microbial membrane proteins, including cytochromes, through hydrogen bonding or electrostatic interactions. These interactions can help construct electron transfer channels between microorganisms, biochar, and pollutants. Extracellular electron transfer (EET) is an important process connecting microorganisms with biochar-based electron-shuttling systems. Direct electron transfer occurs through close contact between microbial cells and biochar surfaces, where microbial electron-transfer proteins, such as outer-membrane cytochromes, facilitate electron transport between microorganisms and conductive carbon domains. Indirect electron transfer is mediated by soluble electron shuttles, extracellular polymeric substances, or redox-active functional groups on biochar surfaces, which reversibly accept and donate electrons. These direct and indirect pathways enable biochar to enhance microbial electron utilization and promote pollutant transformation. In cypermethrin-contaminated soil, the combined application of biochar and Bacillus cereus enhanced cypermethrin degradation and improved soil physicochemical and biological properties [22]. Under anaerobic conditions, biochar promoted the reductive dechlorination of pentachlorophenol by Geobacter sulfurreducens, indicating that biochar can facilitate electron transfer from microorganisms to chlorinated pollutants [23]. When S-ZVI@biochar was combined with dechlorinating bacteria and Shewanella oneidensis MR-1, the degradation pathway was shortened, the electron transfer rate increased, and pollutant degradation efficiency improved, which was attributed to the directional transfer of electrons within the biochar-mediated system [24]. Similarly, the combination of biochar with mixed microbial systems shortened the degradation time of trichloroethylene and enhanced dechlorination efficiency. In phenol wastewater remediation, the current density of the coupled biochar-microorganism system reached 2.5 mA/cm^2^, which was approximately twice that of the single microbial system, further demonstrating the role of biochar in promoting electron transfer within microbial respiratory processes [25].

(3) Synergistic removal of heavy metals and ammonium nitrogen by the composite system

For inorganic pollutants, the biochar-microorganism system often works through the combined effects of adsorption, oxidation, precipitation, and biological transformation. Biochar can enrich metal ions and ammonium near its surface through adsorption, while microorganisms can further promote oxidation, precipitation, or nitrogen transformation. For example, corn straw biochar was used to immobilize Acinetobacter sp. for mine water treatment, achieving removal rates of 98.5%, 95.2%, and 85.3% for Fe^2+^, Mn^2+^, and NH_4_^+^-N, respectively [26]. In this system, biochar adsorbed metal ions and provided attachment sites for microorganisms, whereas microbial activity promoted Fe^2+^ and Mn^2+^ oxidation and precipitation, as well as ammonium conversion through nitrification. This adsorption-bio-oxidation synergy indicates that biochar-microorganism systems can improve the remediation of inorganic pollutants by coupling pollutant enrichment with microbial transformation.

(4) Ternary synergy of plant-microorganism-biochar

**Figure 3:**
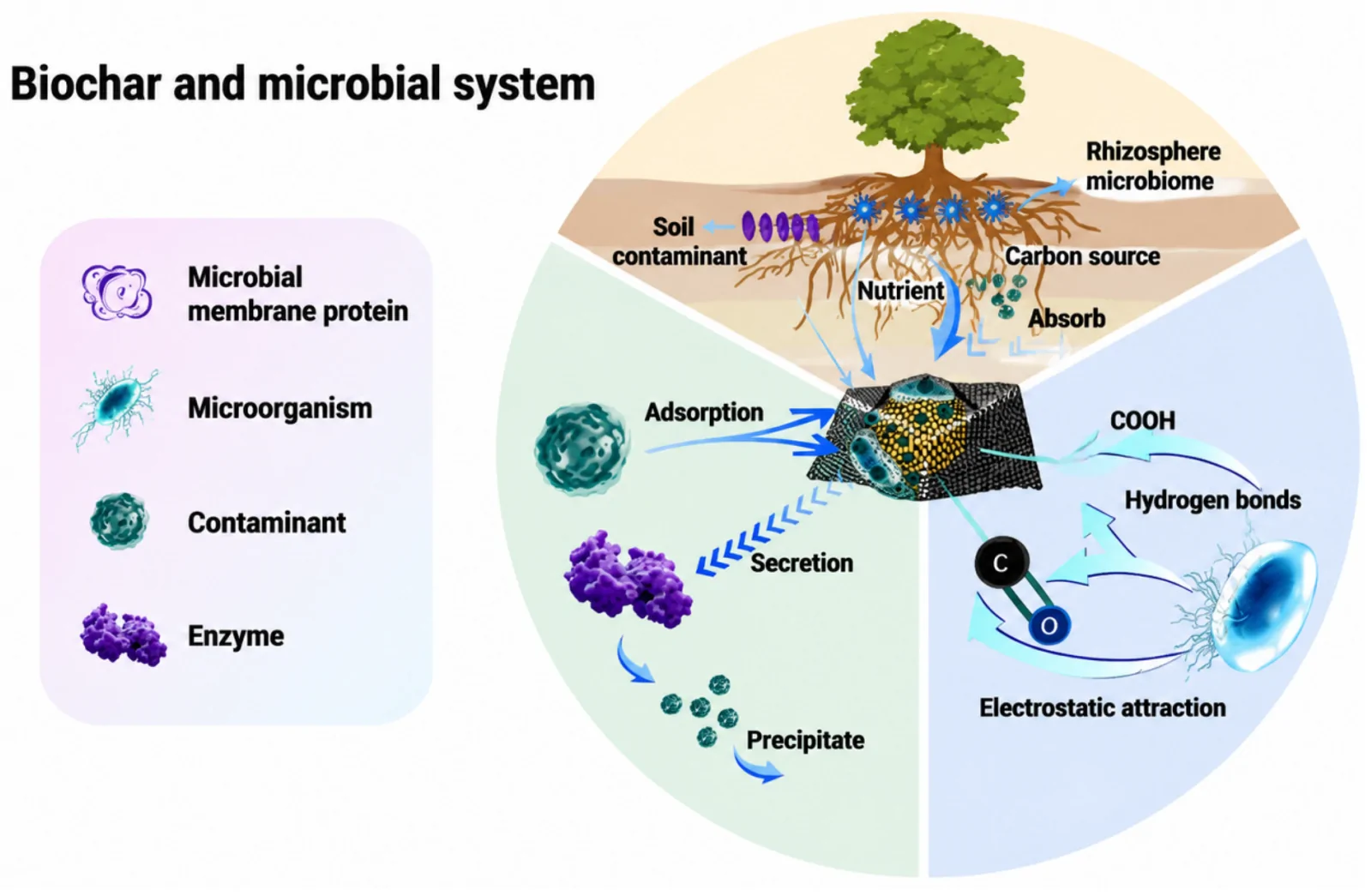
Biochar-immobilized microbial system illustrating adsorption, microbial interaction, and interfacial electron transfer processes.

More complex remediation systems can be formed by integrating biochar, plant-growth-promoting bacteria, and plants. In such ternary systems, biochar can adsorb pollutants, provide habitats for microorganisms, and serve as a medium for electron transfer. Plant roots supply carbon sources and nutrients that support microbial growth and metabolism, while plant-growth-promoting bacteria enhance plant tolerance and participate in pollutant degradation or immobilization. The coordinated interaction among biochar, microorganisms, and plants can therefore improve remediation efficiency in organically contaminated soils (Figure 3) [27]. Compared with single-component systems, the ternary system provides a more stable microenvironment and strengthens the contact among pollutants, microorganisms, and electron-active biochar surfaces, which is beneficial for long-term soil remediation.

### 2.3. Biochar and Nanostructures

**Figure 4:**
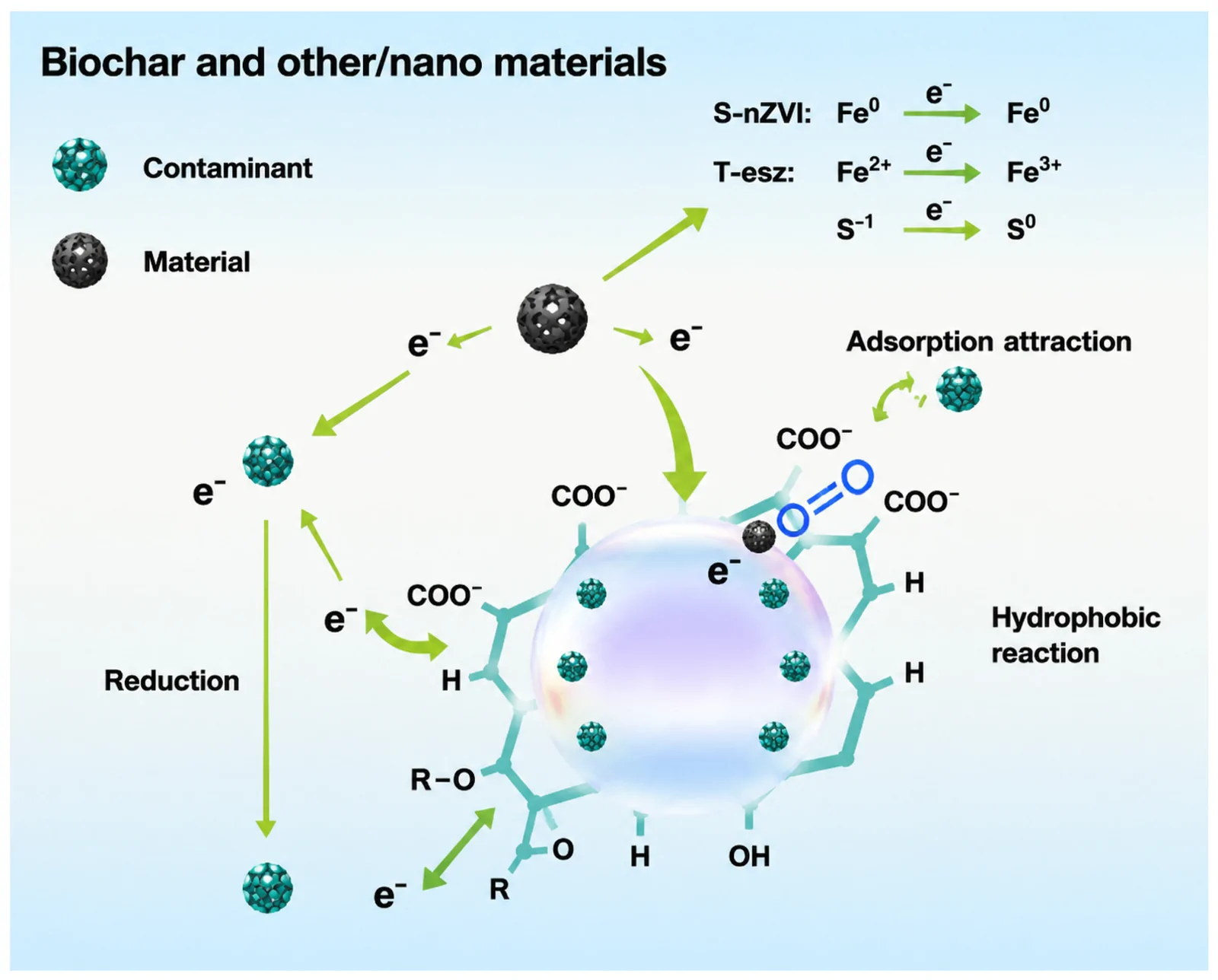
Biochar-based composite system with metal oxides or nanomaterials showing enhanced interfacial electron-transfer pathways.

Nanomaterials have been widely used in environmental remediation because of their high specific surface area, abundant reactive sites, and strong surface activity. However, bare nanomaterials often suffer from aggregation, limited stability, and potential environmental risks, which may restrict their practical application. Combining nanomaterials with biochar provides an effective strategy to overcome these limitations. Biochar can serve as a porous support to disperse nanomaterials, expose more active sites, and provide conductive pathways for interfacial electron transfer. Meanwhile, nanomaterials can introduce electron-rich sites and reactive interfaces that interact with the π–π conjugated structure of biochar, thereby accelerating electron-transfer processes during pollutant transformation (Figure 4) [28].

The biochar–nanomaterial composite system can enhance pollutant degradation by improving interfacial electron transfer. In such systems, biochar does not function merely as a passive support; it can also act as an electron shuttle that facilitates electron exchange between nanomaterials and pollutants through coupled interfacial pathways. For example, in sulfurized nano zero-valent iron supported by biochar (S-nZVI/BC), biochar facilitates electron transfer from zero-valent iron to pollutants, while sulfur modification regulates the distribution of surface sulfur species and improves electron utilization efficiency [29]. This synergistic effect can enhance pollutant reduction and reduce the loss of reactive electrons during the remediation process.

The performance of biochar-supported nanomaterials is closely related to the pore structure of biochar and the loading amount of nanomaterials. Hierarchical pore structures can improve the dispersion of nanomaterials and shorten the diffusion distance between pollutants and reactive sites. At the same time, an appropriate mass ratio of nanomaterials to biochar is necessary to balance active-site exposure, electron transfer efficiency, and material stability. Therefore, regulating biochar pore structure and nanomaterial loading is an important approach for optimizing the electron-shuttling capability and pollutant transformation efficiency of biochar-nanomaterial composites [30].

### 2.4. Other Composite Structures

The combination of biochar with transition metal compounds or photocatalytic materials can significantly expand its application scope in environmental remediation and generate multifunctional composite structures. The synergistic mechanisms depend on the properties of the composite materials. When biochar is combined with transition metal compounds such as FeS_2_ or Fe_3_O_4_, the metals can act as electron donors to reduce pollutants through valence-state changes. For instance, in ball-milled biochar-iron sulfide composites (BM-FeS_2_@BC), Fe(II)/S(-II) in FeS_2_ serves as an electron donor, reducing Cr(VI) to Cr(III) with biochar as a mediator. The porous structure of biochar also adsorbs the precipitation products, leading to high Cr(VI) removal efficiency [31].

Biochar-magnetic composites, such as Fe_3_O_4_@BC, combine the advantages of easy magnetic separation and enhanced electrical conductivity, which promote electron transfer and significantly improve the aerobic biodegradation of gaseous pollutants such as p-xylene [32]. In biochar-supported photocatalyst systems, such as rice husk activated carbon-supported TiO_2_ (AC@TiO_2_), biochar functions as an electron acceptor, inhibiting the recombination of photogenerated electrons and holes. This effect enhances photocatalytic degradation performance, resulting in 23% and 47% increases in the degradation efficiencies of reactive red and ofloxacin, respectively, compared with pure TiO_2_ [33].

The performance of these composite systems can be further optimized by regulating the crystal planes of metal compounds, such as the (100) plane of FeS_2_, or by modifying the electronic structure of biochar, for example, through nitrogen doping [34]. These strategies can enhance interfacial electron transfer efficiency, thereby improving pollutant degradation. Table 2 summarizes relevant studies on pollutant removal using biochar combined with various microorganisms or materials.

Overall, biochar-based composite structures, including transition metal and photocatalyst hybrids, provide a versatile platform for enhanced environmental remediation through synergistic electron transfer mechanisms and adsorption-degradation processes.

However, the relationship between biochar properties and electron transfer performance is influenced by multiple factors and cannot be explained by a single structural characteristic. For example, higher pyrolysis temperatures can promote aromatic carbon formation and improve electron transport, but may also reduce oxygen-containing functional groups involved in reversible electron exchange. Therefore, differences in feedstock composition and preparation conditions may contribute to variations in reported electron-shuttling capabilities among different studies.

In addition, the practical application of biochar-supported composite systems requires further consideration of environmental safety and long-term stability. The potential release of metal-containing components or nanoparticles under complex environmental conditions should be evaluated to avoid secondary risks. Moreover, regeneration capacity, interfacial stability, and economic feasibility need to be systematically investigated before large-scale application of these composite materials. Although enhanced pollutant transformation performance is frequently associated with biochar-mediated electron transfer, direct evidence of electron-shuttling behavior requires specific electrochemical or redox characterization. In many reported studies, improved remediation efficiency may result from the combined effects of adsorption enrichment, microbial interactions, surface reactions, and electron-transfer regulation. Therefore, electron-transfer contributions should be interpreted based on available experimental evidence rather than solely inferred from overall removal performance.

## 3. Mechanisms of Biochar as an Electron Shuttle in Pollutant Remediation

**Figure 5:**
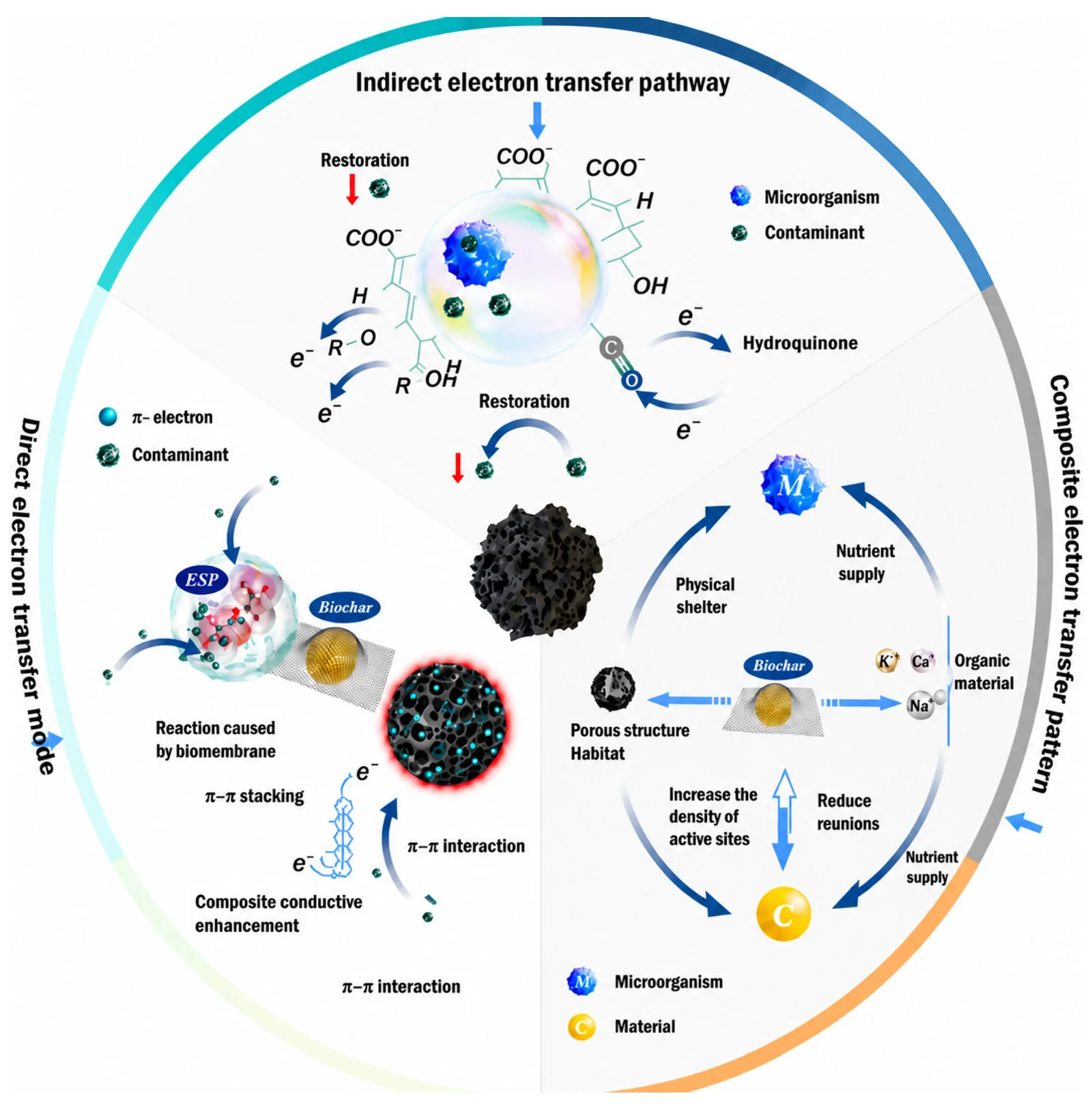
Sector-based conceptual representation of three biochar-mediated electron-transfer pathways: direct electron transfer through conductive carbon structures in the left sector, indirect electron transfer mediated by redox-active surface functional groups in the upper sector, and composite interfacial electron transfer involving microorganisms, minerals, and functional materials in the right sector.

Based on the biochar-based interfacial systems described above, biochar-mediated electron transfer is governed by conductive carbon structures, redox-active surface sites, external electron donors or acceptors, and interfacial interactions with pollutants or other functional components. Depending on the predominant electron-transfer route, three conceptual pathways are presented in Figure 5. The left, upper, and right sectors represent direct electron transfer through conductive carbon structures, indirect electron transfer mediated by reversible redox-active surface groups, and composite interfacial electron transfer involving microorganisms, minerals, or other functional materials, respectively. These pathways are not mutually exclusive and may coexist in biochar-based systems. Their identification requires multiple experimental approaches rather than inference from pollutant-removal performance alone. Electrical conductivity measurements and electrochemical impedance spectroscopy evaluate conductive properties and interfacial charge-transfer resistance, whereas cyclic voltammetry and EDC, EAC, and EEC analyses provide evidence of reversible redox activity and electron-storage capability.

The performance of these pathways depends on the balance between conductive carbon domains and redox-active surface sites. Increased pyrolysis temperature may enhance carbonization and conductivity while reducing oxygen-containing functional groups; therefore, biochar electron-shuttling capability is determined by the combined effects of conductive structures, redox-active sites, and interfacial interactions rather than by a single structural parameter.

### 3.1. Direct Electron Transfer Model: Conductive Carbon Network Mechanism

Direct electron transfer is primarily governed by the conductive carbon matrix formed during high-temperature pyrolysis. Graphitized carbon domains provide continuous pathways for electron migration through delocalized π–electron systems, thereby enabling long-range interfacial electron transfer across biochar-based interfaces. The contribution of conductive carbon networks to electron transfer can be evaluated through electrical conductivity measurements and electrochemical impedance spectroscopy (EIS), where enhanced conductivity and reduced charge-transfer resistance indicate improved electron transport capability.

Electron transfer occurs through π–π interactions between aromatic pollutants and graphitized carbon structures, where these interactions primarily enhance interfacial contact and reduce electron-transfer distances, thereby facilitating subsequent electron transfer processes [36,37]. In addition, when biochar is coupled with conductive materials such as MXene (Ti_3_C_2_T_x_), an extended interfacial electron-conduction network is formed, significantly improving interfacial electron-transfer efficiency [38].

The porous structure of biochar facilitates pollutant enrichment near reactive sites through physical adsorption and π–π interactions, thereby increasing the probability of interfacial electron transfer. Moreover, biochar provides colonization surfaces for anaerobic microorganisms (e.g., electrogenic and dehalogenating bacteria), promoting biofilm formation and strengthening biochar-mediated electron-transfer coupling between microorganisms and pollutants [39]. In nitrogen transformation systems, biochar regulates interfacial electron flow, thereby enhancing nitrate reduction efficiency. In iron-containing systems, electron transfer efficiency is further improved through coupling between the carbon matrix and Fe^2+^/Fe^3+^ redox cycles [40]. The efficiency of direct electron transfer is closely related to the development of conductive carbon domains and their structural connectivity, which facilitate electron migration across biochar interfaces. However, this pathway is also influenced by carbonization degree and the accessibility of conductive regions.

### 3.2. Indirect Electron Transfer Mode: Surface Functional Group-Mediated Redox Process

Indirect electron transfer is driven by redox-active surface functional groups, including hydroxyl (-OH), carboxyl (-COOH), carbonyl (C=O), and phenolic groups. These functional groups act as electron mediators through reversible interfacial redox cycling processes. The reversible redox cycling of quinone/hydroquinone-like structures and other oxygen-containing groups enables electron donation and acceptance during pollutant transformation. The reversible transformation between carbonyl and hydroxyl groups (C=O↔-OH) and related redox transitions among oxygen-containing functional groups enable electron transfer to redox-sensitive pollutants such as U(VI), promoting its reduction to U(IV) [38]. In addition, redox transitions among hydroxyl, carbonyl, and carboxyl groups contribute to continuous interfacial electron exchange within aromatic carbon frameworks [41]. Quinone-like structures, carbonyl groups, and other oxygen-containing moieties can act as electron acceptors or donors during pollutant transformation. However, the contribution of these redox-active sites depends on their abundance, surface accessibility, and interaction with surrounding environmental components.

Phenolic groups can be oxidized to phenoxy radicals and further transformed into quinone structures, which participate in interfacial electron-accepting processes [42]. Among these functional groups, phenolic moieties primarily act as electron donors, whereas quinone structures function as electron acceptors.

The electron exchange capacity (EEC), defined as the sum of electron-donating capacity (EDC) and electron-accepting capacity (EAC), is strongly dependent on the abundance of surface redox-active groups, particularly in low-temperature biochar [43]. Cyclic voltammetry (CV) analysis can further reveal reversible redox peaks associated with these functional groups, providing electrochemical evidence for their electron-storage and electron-transfer capability. These properties regulate the efficiency of indirect electron-transfer processes during pollutant transformation.

### 3.3. Composite Electron Transfer Model: Interfacial Coupling Mechanism

The composite electron transfer pathway arises from interfacial coupling among biochar, pollutants, microorganisms, and coexisting materials, with biochar providing a heterogeneous platform for charge redistribution. In these systems, biochar can act as an electron mediator by providing conductive pathways and redox-active sites that facilitate electron transfer between microorganisms, minerals, or other functional materials. The overall electron-transfer efficiency is therefore determined by the interfacial compatibility, contact area, and electronic interactions between different components. The enhancement of interfacial electron transfer in composite systems can be evaluated through electrochemical characterization, such as electrochemical impedance spectroscopy (EIS), conductivity measurements, and redox activity analysis, which provide evidence for improved charge transport and interfacial electron exchange.

Biochar concentrates pollutants on its surface or within its pore structure, increasing the probability of interfacial reactions and electron exchange. Meanwhile, surface functional groups and conductive carbon domains jointly regulate electron distribution at the interface [44]. When biochar is combined with metal oxides or nanomaterials such as Fe_3_O_4_ or α-Fe_2_O_3_, nanoparticles are dispersed on the biochar surface, reducing aggregation and increasing the exposure of reactive redox-active sites [45]. This interfacial coupling enhances electron-transfer efficiency and pollutant transformation performance.

Overall, composite systems enhance interfacial electron transfer efficiency through adsorption enrichment, interfacial contact regulation, and charge redistribution. For example, Fe_3_O_4_–biochar composites exhibit higher pollutant transformation efficiency compared with individual components [46], suggesting that interfacial interactions between different components can contribute to enhanced electron-transfer-assisted transformation. However, distinguishing electron transfer contributions from other simultaneous processes remains challenging in complex remediation systems.

### 3.4. Experimental Approaches for Evaluating Biochar Electron-Shuttling Capability

Although the electron-shuttling function of biochar has been widely proposed in environmental remediation systems, direct experimental evidence is required to distinguish electron transfer processes from adsorption, surface complexation, and other physicochemical interactions [47]. Therefore, a combination of structural characterization, electrochemical analysis, and redox-capacity measurements has been applied to evaluate the electron transfer properties of biochar. Because different electron-transfer pathways may coexist in biochar-based systems, no single analytical technique can independently confirm the electron-shuttling mechanism.

Electrical conductivity measurements are commonly used to evaluate the ability of carbon structures within biochar to facilitate electron migration. The formation of aromatic carbon domains and graphitic structures during pyrolysis can improve electron transport through conjugated carbon networks. However, conductivity alone cannot fully represent electron-shuttling capability because electron shuttles require reversible electron acceptance and donation rather than simple charge transport. Therefore, conductivity measurements are usually combined with structural analyses, such as Raman spectroscopy, X-ray photoelectron spectroscopy (XPS), and elemental analysis, to clarify the contribution of carbon structures and surface chemistry to electron transfer behavior [47].

Electrochemical techniques, including electrochemical impedance spectroscopy (EIS) and cyclic voltammetry (CV), provide supporting evidence for interfacial electron transfer processes [11]. EIS analysis is generally used to evaluate charge-transfer resistance at biochar interfaces. A lower charge-transfer resistance indicates reduced resistance to electron migration and provides supporting evidence for enhanced interfacial electron transfer processes. CV analysis can identify reversible redox reactions associated with electron-active functional groups, such as quinone/hydroquinone couples and oxygen-containing groups. The presence of reversible oxidation–reduction peaks suggests that biochar possesses electron exchange sites capable of participating in redox cycling. Thus, EIS mainly reflects electron-transport characteristics, whereas CV provides evidence of reversible redox activity; these results should be interpreted in combination rather than independently.

Electron exchange capacity (EEC), including electron donating capacity (EDC) and electron accepting capacity (EAC), provides quantitative information on the redox activity of biochar [47,48]. EDC represents the ability of biochar to release electrons, whereas EAC reflects its capacity to accept electrons. The coexistence of electron donation and electron acceptance abilities allows biochar to act as an electron buffer and mediator in microbial respiration, mineral reduction, and pollutant transformation processes. Compared with conductivity measurements, EEC, EDC, and EAC better describe the reversible electron transfer capability required for electron-shuttling behavior.

Persistent free radicals are another electron-active component associated with biochar redox activity. Electron paramagnetic resonance (EPR) spectroscopy has been applied to identify carbon-centered and oxygen-centered radicals generated during pyrolysis [47]. These radicals may participate in reversible electron transfer reactions and contribute to the redox properties of biochar. However, the formation and stability of persistent free radicals depend on feedstock characteristics, pyrolysis conditions, and environmental aging, and therefore their contribution should be evaluated together with electrochemical and redox-capacity measurements.

It should be noted that enhanced pollutant removal efficiency alone cannot be directly interpreted as evidence of electron-shuttling behavior. In biochar-based remediation systems, adsorption, surface complexation, microbial activity enhancement, and electron transfer processes may occur simultaneously. Therefore, combining electrochemical measurements, redox-capacity analysis, and pollutant transformation characterization is necessary to determine the contribution of electron transfer mechanisms in different environmental systems.

## 4. Application of Biochar-Mediated Electron Shuttling in Soil Pollutant Remediation

Biochar-based systems act as interfacial electron-transfer platforms that integrate adsorption, redox transformation, and microbial interactions to regulate the mobility, speciation, and transformation of soil pollutants. The remediation performance is governed by coupled interactions among biochar, pollutants, microorganisms, and coexisting mineral or nanomaterial phases, which collectively determine interfacial electron distribution, redox reactivity, and pollutant fate.

### 4.1. Heavy Metal Remediation

Heavy metals are persistent contaminants because they cannot be mineralized and may undergo continuous transformation among different chemical forms in environmental systems. Their remediation by biochar involves multiple mechanisms, including adsorption, ion exchange, surface complexation, precipitation, and, for redox-active metals, electron-transfer-assisted transformation. Therefore, the contribution of electron-shuttling processes depends on the chemical properties and redox characteristics of specific metals. For non-redox-active metals, such as Cd(II) and Pb(II), biochar immobilization is mainly controlled by adsorption, complexation, ion exchange, and precipitation processes. In contrast, for metals with variable oxidation states, such as Cr(VI), electron transfer can directly participate in changing metal speciation and mobility through reduction reactions. Thus, electron shuttling should be considered as one component of coupled remediation processes rather than the sole mechanism responsible for heavy metal immobilization.

In practical remediation systems, these mechanisms rarely occur independently, and their relative contributions depend on biochar properties, pollutant characteristics, and environmental conditions. Therefore, distinguishing the relative contribution of different mechanisms is essential for evaluating the role of biochar-mediated electron transfer in pollutant remediation.

#### 4.1.1. Remediation of Cadmium

Cadmium is a typical non-redox-active heavy metal; therefore, its immobilization is mainly governed by adsorption, ion exchange, and precipitation rather than a direct electron-transfer process. Biochar contributes to Cd immobilization by providing abundant oxygen-containing functional groups (e.g., -COOH, -OH), which enhance surface complexation with Cd(II). In addition, biochar-induced pH elevation in soil promotes the formation of Cd(OH)_2_, carbonates, and other low-solubility species, thereby reducing Cd mobility [49].

In biochar-microorganism systems, microbial metabolic activity modifies the surrounding microenvironment (e.g., pH and dissolved organic matter concentration), indirectly enhancing Cd adsorption and precipitation processes [21]. In biochar-g-C_3_N_4_ systems, nitrogen-containing sites (e.g., pyridinic N) and sulfur-related functional groups contribute to Cd stabilization via coordination interactions, forming stable complexes such as Cd-S-C structures [50].

Overall, Cd immobilization is dominated by adsorption–precipitation and coordination processes, while electron transfer effects are indirect and mainly act through interfacial chemical regulation rather than direct redox transformation.

#### 4.1.2. Mercury Remediation

Mercury exhibits variable redox behavior in environmental systems; therefore, its remediation may involve both adsorption and electron-transfer-mediated redox transformation under specific conditions.

Biochar immobilizes mercury through complexation with oxygen-containing functional groups (such as hydroxyl and carboxyl groups) and π-conjugated aromatic structures, which provide abundant binding sites and reduce Hg bioavailability and mobility [51]. These interactions also influence electron redistribution at the biochar-contaminant interface.

More importantly, biochar can function as an interfacial electron transfer mediator, enabling partial reduction of Hg(II) to Hg(0) under specific redox conditions. This process is driven by electron redistribution within the carbon matrix and redox cycling of surface-active functional groups.

In biochar-based composite systems, such as graphene–biochar materials, the presence of a conductive graphene network further enhances electron transport efficiency. The π-electron conjugated structure of graphene provides additional conductive pathways, promoting interfacial electron migration and improving mercury transformation efficiency [52]. This coupling between graphene and biochar enhances both adsorption capacity and electron transfer efficiency.

Overall, mercury remediation by biochar involves coupled mechanisms of adsorption, surface complexation, and electron-transfer-mediated redox transformation. Compared with non-redox metals such as Cd, Hg exhibits a stronger dependence on electron-shuttling processes, making it a representative system for studying biochar-mediated redox transformation mechanisms in environmental systems.

#### 4.1.3. Chromium Remediation

Chromium contamination has become increasingly severe due to industrial activities. In the environment, chromium mainly exists in two oxidation states, Cr(III) and Cr(VI), among which Cr(VI) is highly soluble, mobile, and toxic. Therefore, its reduction to Cr(III) is a key process for detoxification and environmental remediation.

Hexavalent chromium [Cr(VI)] is a strong electron acceptor, and its transformation to Cr(III) involves electron transfer processes in soil systems. Biochar plays a critical role in this process by acting as an electron shuttle that mediates electron transfer between electron donors and Cr(VI). The electron-rich carbon matrix and surface functional groups of biochar enable interfacial electron redistribution, thereby facilitating Cr(VI) reduction [53].

In biochar-iron-based systems, such as nanoscale zero-valent iron (nZVI) composites, Fe^0^ acts as a strong electron donor, while biochar functions as a conductive matrix that enhances electron transport and suppresses nanoparticle aggregation. This synergistic interaction improves electron utilization efficiency and significantly enhances Cr(VI) reduction performance [54].

Similarly, in biochar–pyrite composites (BM-FeS_2_@BC), Fe(II)/S(-II) species serve as electron donors, while biochar mediates electron transfer toward Cr(VI), promoting its reduction to Cr(III). In addition, the porous structure of biochar facilitates the immobilization of Cr(III) precipitation products, contributing to overall pollutant stabilization [31].

Overall, Cr(VI) remediation represents a typical electron-shuttling-assisted process in biochar-based systems. Compared with other heavy metals, Cr(VI) exhibits the strongest dependence on interfacial electron transfer, making it a representative model for elucidating biochar-mediated redox transformation mechanisms in soil environments.

#### 4.1.4. Uranium Remediation

Uranium [U(VI)] is a long-lived radioactive contaminant widely associated with nuclear industry activities. It exhibits high mobility in aqueous environments, particularly under acidic conditions, where it exists in soluble forms and can migrate through groundwater systems. Therefore, its immobilization and reduction are essential for environmental risk mitigation.

Biochar can immobilize U(VI) through a combination of adsorption, ion exchange, and redox-related surface processes. The abundance and type of surface functional groups strongly influence uranium retention and transformation. For instance, biochar derived from macauba nut shells exhibits temperature-dependent surface chemistry, where biochar produced at 350 °C contains abundant oxygen-containing functional groups, leading to enhanced U(VI) removal via coupled ion exchange and surface-mediated reduction processes [55]. In this context, electron transfer between biochar surface functional groups and U(VI) contributes to partial reduction toward less mobile forms.

In biochar-based composite systems, the immobilization efficiency of U(VI) can be significantly improved. Silicon-containing biochar loaded with iron oxide nanoparticles provides enhanced structural stability and a higher density of reactive sites. The synergistic interaction between silicon-modified surfaces and metal oxides promotes interfacial adsorption and electron redistribution, achieving high U(VI) removal efficiency under optimal pH conditions [56]. This suggests that both surface complexation and electron-transfer-assisted processes contribute to uranium immobilization.

Advanced biochar-based composites, such as Ti_3_C_2_T_x_@biochar–PDA/PEI, further enhance U(VI) removal through combined mechanisms including electrostatic attraction, ion exchange, and surface chelation [38]. In such systems, the conductive component improves interfacial charge distribution, indirectly facilitating electron-transfer-related processes.

In addition, electrocatalytic systems based on biochar-derived materials have demonstrated the ability to remove U(VI) from aqueous environments through redox reactions. For example, Fe–N–C catalysts can extract U(VI) via electrocatalytic processes, where in situ spectroscopic analysis reveals intermediate species involved in electron transfer pathways [37]. These findings indicate that biochar-based materials can participate in both adsorption-driven and electron-transfer-assisted uranium remediation processes.

Overall, uranium remediation represents a multi-pathway process involving adsorption, surface complexation, and electron-transfer-assisted reduction. Compared with Cd and Hg, U(VI) exhibits a more complex dependency on coupled interfacial and redox processes, making it an important system for understanding electron-shuttling behavior in biochar-based environmental remediation.

#### 4.1.5. Restoration of Other Heavy Metals

In addition to Cd, Hg, Cr, and U, biochar is also widely applied for the remediation of other heavy metals such as Pb, Zn, Cu, and Ni. These metals generally exhibit weak or non-redox-active behavior in soil environments, and their immobilization is mainly governed by adsorption, surface complexation, and precipitation processes, with limited direct involvement of electron transfer reactions.

Biochar can effectively reduce the mobility and bioavailability of these metals through its porous structure and abundant surface functional groups. Oxygen-containing functional groups such as hydroxyl, carboxyl, and phenolic groups provide active binding sites for metal ion complexation, while the porous structure enhances physical adsorption and ion exchange capacity.

In biochar-based composite systems, such as biochar combined with iron oxides, the immobilization efficiency of heavy metals can be further improved. Iron oxides provide additional active sites for surface complexation, while biochar enhances dispersion and improves interfacial contact, thereby increasing the stability of immobilized metal species [57].

In addition, biochar amendment can modify soil physicochemical properties, including increasing pH and organic matter content, which promotes the precipitation of metal hydroxides or carbonates and further reduces metal mobility. These changes enhance the overall immobilization performance of Pb, Zn, Cu, and Ni in soil systems.

**Figure 6:**
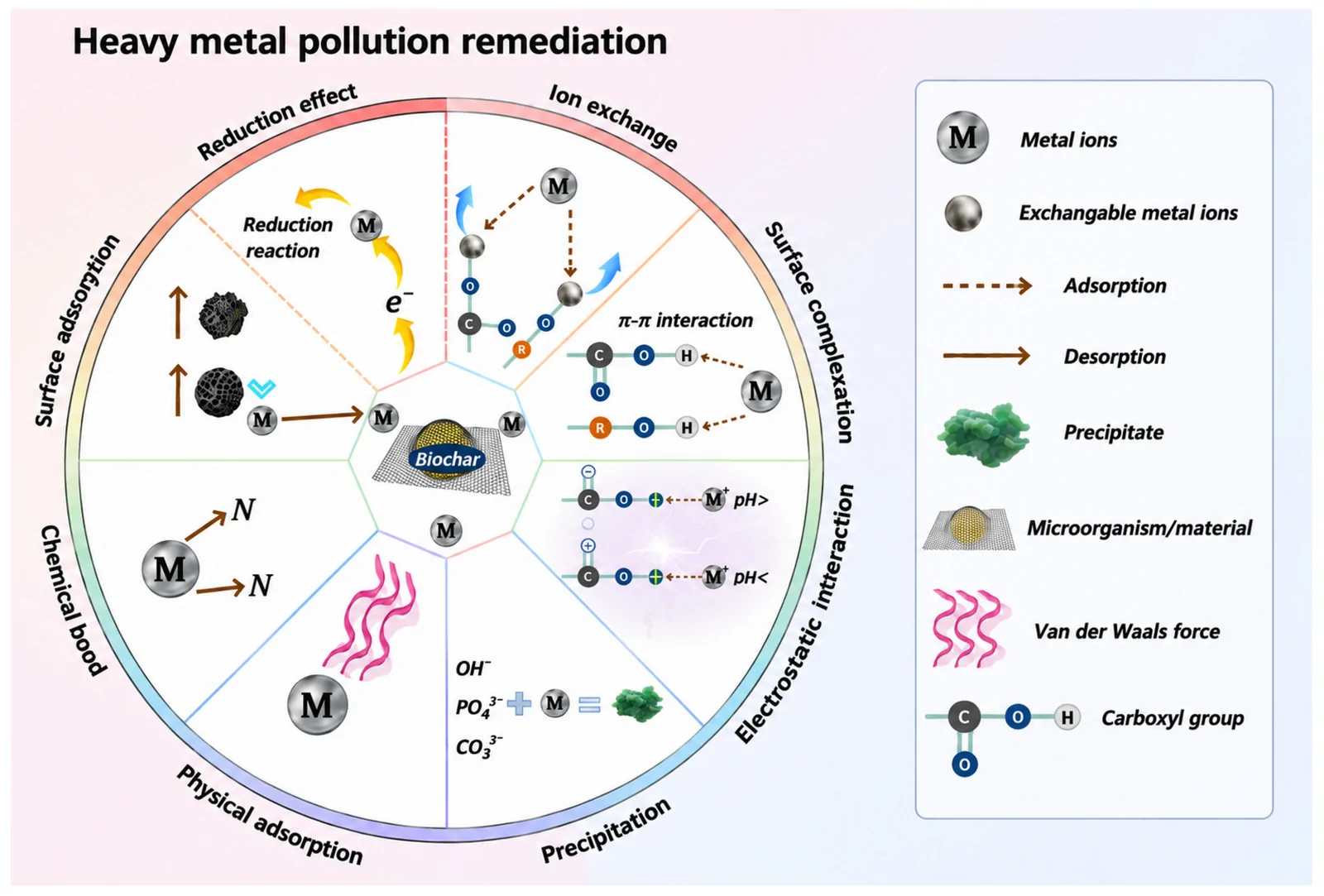
Mechanistic illustration of heavy metal immobilization via adsorption, complexation, and interfacial redox reactions.

Overall, the remediation of these metals is primarily controlled by adsorption–complexation–precipitation mechanisms, with only minor contributions from electron-transfer-related interfacial effects. Compared with Cr(VI) and U(VI), these metals exhibit significantly weaker dependence on electron-shuttling processes, indicating that biochar-mediated electron transfer plays a secondary role in their immobilization (Figure 6).

### 4.2. Organic Pollutant Remediation

Organic pollutants are widely distributed in agricultural and industrial soils and represent a persistent environmental challenge due to their toxicity and resistance to natural degradation. Their transformation in soil systems is closely associated with microbial activity and interfacial electron transfer processes. Biochar enhances the remediation of organic pollutants through coupled adsorption enrichment, microbial degradation, and electron-transfer-assisted transformation processes. Table 3 summarizes representative biochar-immobilized microorganism/material systems and their reported remediation performance for heavy metals, organic pollutants, nutrient pollutants, and co-contaminated systems.

#### 4.2.1. Remediation of Pesticide and Antibiotic Contamination

Pesticides and antibiotics represent major classes of organic pollutants in soil environments. Pesticides include organochlorine compounds (e.g., DDT and BHC), organophosphorus compounds (e.g., dichlorvos and dimethoate), and pyrethroid compounds (e.g., cypermethrin), which differ in toxicity, persistence, and environmental behavior [58]. These compounds can persist in soils due to their low biodegradability and may exert long-term ecological risks.

Biochar enhances the remediation of these pollutants through coupled adsorption and microbial degradation processes, in which biochar-mediated electron-transfer interactions may further influence microbial transformation efficiency. The porous structure of biochar first facilitates the enrichment of organic pollutants on its surface, thereby increasing their accessibility to microbial degradation and subsequent interfacial transformation. More importantly, biochar functions as an interfacial electron mediator that connects microbial cells and adsorbed pollutant molecules, forming a bacteria-biochar-pollutant electron-transfer pathway that accelerates biodegradation processes.

For example, in cypermethrin-contaminated soil, the combined application of biochar and Bacillus cereus significantly enhanced degradation efficiency [22]. This improvement is associated with the conductive carbon structure of biochar, which can facilitate interfacial electron transfer between microbial cells and adsorbed pollutants, thereby enhancing microbial transformation processes.

Similarly, in atrazine-contaminated systems, biochar loaded with Paenarthrobacter sp. AT5 enhances degradation efficiency and alleviates phytotoxic effects [59]. This process is associated with redox-active surface functional groups of biochar, particularly quinone-like structures, which participate in reversible electron-transfer cycles and regulate microbial metabolic activity, thereby promoting pollutant degradation.

**Figure 7:**
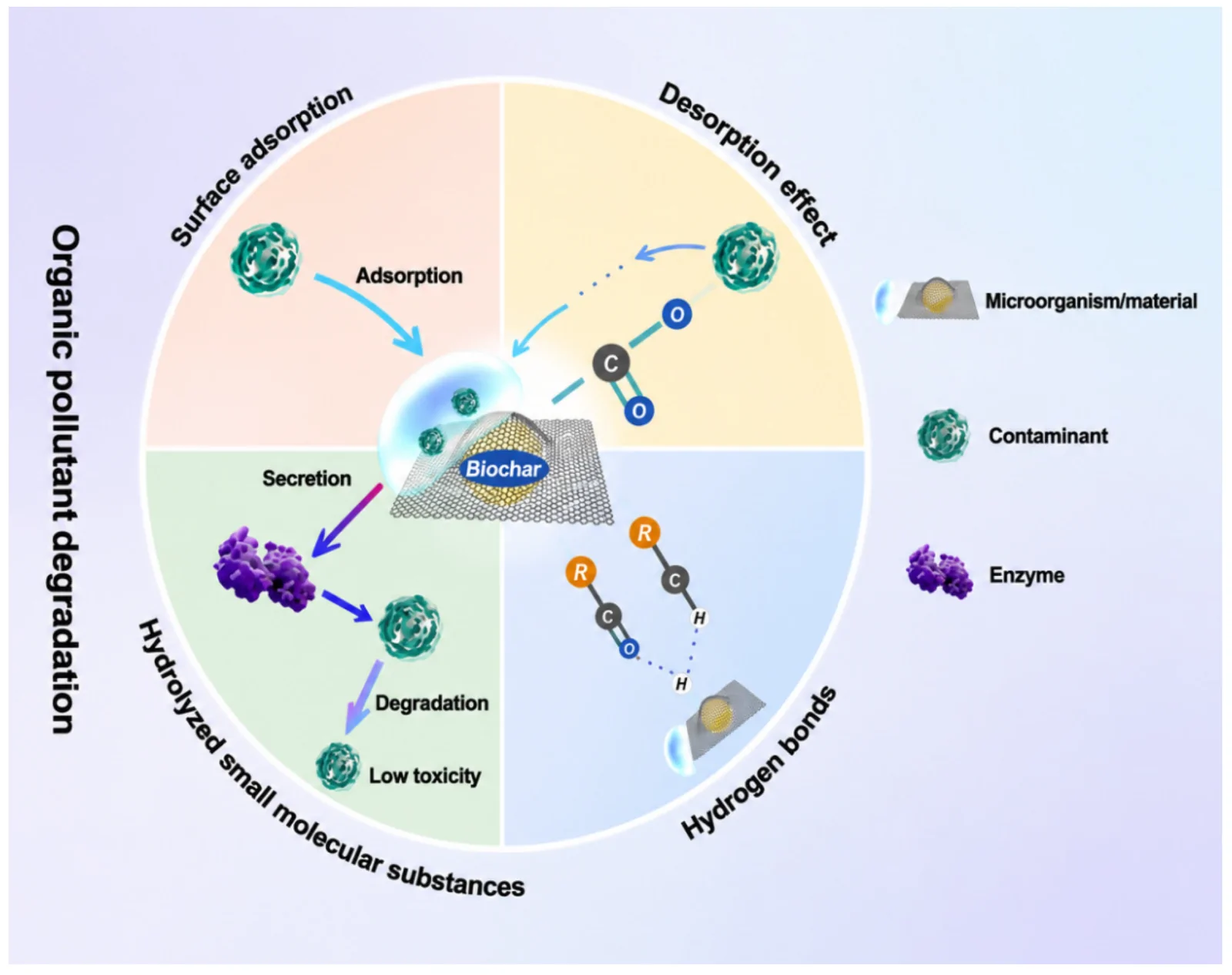
Schematic of organic pollutant degradation mediated by adsorption enrichment and electron-transfer-assisted transformation.

Overall, pesticide and antibiotic remediation by biochar involves coupled adsorption and microbial degradation processes, where biochar-mediated electron-transfer interactions can enhance interfacial communication between biochar, microorganisms, and pollutants. Biochar functions as an electron mediator and conductive pathway, improving pollutant accessibility and facilitating biodegradation efficiency (Figure 7).

#### 4.2.2. Remediation of Other Organic Pollutants

Polybrominated diphenyl ethers (PBDEs), as persistent brominated flame retardants, are widely distributed in consumer products such as electronics and furniture. Due to their high hydrophobicity and chemical stability, they tend to accumulate in soil environments and pose long-term ecological risks. Biochar derived from biomass sources such as water hyacinth contains abundant oxygen-containing functional groups, including quinone and carboxyl groups, which can enhance the microbial degradation of PBDEs such as BDE-47 [60] by regulating interfacial redox interactions and microbial activity.

In biochar-microorganism systems, pollutant transformation is mainly governed by coupled interfacial processes involving electron transfer and microbial metabolism. Biochar acts as an electron mediator that facilitates electron exchange between microbial cells and adsorbed PBDE molecules, forming a microorganism-biochar-pollutant electron-transfer pathway. This electron-shuttling effect can promote the reductive transformation of PBDEs, highlighting the role of biochar as an interfacial electron-transfer component rather than merely a passive adsorbent.

Phthalates (PAEs), particularly bis(2-ethylhexyl) phthalate (DEHP), are widely used plasticizers that are frequently detected in soil environments due to their large-scale production and persistence. Biochar-based immobilization systems have been shown to enhance DEHP degradation efficiency. For instance, biochar-immobilized microorganisms achieved a degradation rate of 76.61% for 50 mg/L DEHP within 120 h, which was significantly higher than that of free microbial systems (62.64%) and biochar alone (9.45%) [61]. This improvement is attributed to the dual function of biochar, which provides microbial attachment sites and facilitates biochar-mediated interfacial electron-transfer interactions, while surface functional groups (e.g., -OH and -COOH) stabilize microbial cells through hydrogen bonding interactions.

Emerging contaminants (ECs), including antibiotics, neonicotinoid pesticides, and nanoplastics, represent a diverse group of synthetic pollutants that are not routinely monitored but pose potential ecological risks. The removal mechanisms of ECs by biochar are generally dominated by micropore filling, surface adsorption, and electrostatic interactions, while electron-transfer processes may contribute to the transformation of specific redox-active contaminants. Importantly, redox-active functional groups such as quinone moieties can also participate in interfacial electron transfer processes and may enhance microbial degradation efficiency [62]. In this context, biochar serves as a multifunctional interface that integrates adsorption enrichment and electron-transfer regulation, thereby promoting the transformation of complex organic contaminants in soil systems. The differences in remediation performance among biochar-based systems are associated with variations in biochar properties, pollutant characteristics, and microbial activity. Therefore, the contribution of electron transfer should be evaluated together with adsorption enrichment and biological transformation, as these processes often jointly determine pollutant fate in biochar-mediated remediation systems.

### 4.3. Multivariate Enrichment Pollutant Remediation

Soils are frequently co-contaminated by multiple classes of pollutants, among which combined contamination by heavy metals/metalloids and polycyclic aromatic hydrocarbons (PAHs) is particularly widespread. PAHs are characterized by long environmental persistence, with soil half-lives ranging from days to hundreds of days, leading to sustained ecological and health risks [63]. In such systems, pollutant interactions and environmental behavior are highly complex, making single-pollutant remediation strategies insufficient.

Biochar exhibits significant advantages in co-contaminated systems by simultaneously regulating the fate of organic and inorganic pollutants through coupled adsorption, microbial interaction, and interfacial electron transfer processes. In these systems, biochar acts as a central interfacial platform that mediates pollutant distribution and electron exchange between different contaminant species.

In metal–organic co-contaminated environments, biochar–microorganism systems can enhance the immobilization of heavy metals such as Cd and Cu. This improvement is associated with the secretion of extracellular polymeric substances (EPS) by microorganisms, which contain abundant functional groups capable of interacting with biochar surfaces. These interactions promote microbial colonization and enhance interfacial interactions, which may facilitate electron exchange at the biochar-microbe interface, thereby strengthening metal immobilization processes [21].

In PAH–metal co-contaminated systems, biochar-immobilized microbial consortia can simultaneously enhance PAH degradation and heavy metal stabilization. Oxygen-containing functional groups on biochar surfaces, such as hydroxyl and carbonyl groups, facilitate electron exchange between microbial cells and pollutants. In this process, biochar functions as an electron mediator that couples microbial metabolic activity with pollutant transformation pathways, enabling coordinated remediation of organic and inorganic contaminants [64].

Overall, co-contaminated systems highlight the role of biochar as an interfacial electron redistribution platform, where adsorption enrichment, microbial interaction, and electron transfer coupling jointly regulate pollutant behavior. Compared with single-pollutant systems, multipollutant contamination remediation relies more strongly on interfacial electron-transfer-mediated processes, enabling enhanced stabilization and transformation of complex soil environments.

### 4.4. Remediation of Nitrogen and Phosphorus Pollution

Nitrogen pollution is primarily derived from agricultural systems, where the use efficiency of synthetic nitrogen fertilizers is generally low. Excess nitrogen is lost through leaching, volatilization, and runoff, contributing to environmental issues such as eutrophication and ecosystem imbalance [65]. In soil systems, nitrogen transformation processes are closely associated with microbial metabolism and electron transfer processes.

Biochar plays an important role in regulating nitrogen cycling by modulating interfacial electron-transfer processes and microbial community structure. In anaerobic nitrogen removal systems, biochar has been shown to enhance multiple nitrogen transformation processes by facilitating electron donation and improving microbial activity through improved electron exchange at biochar-microorganism interfaces. For example, in the anaerobic ammonium oxidation (anammox) system, biochar promotes nitrogen removal through the coupling of multiple pathways, including anammox, denitrification, and Feammox processes, thereby significantly improving total nitrogen removal efficiency [66]. This enhancement is attributed to the ability of biochar to regulate electron flow and optimize microbial electron utilization efficiency.

In addition to nitrogen transformation systems, biochar can also interact with functional microbial consortia in soil environments. In biochar-effective microorganism (EM) systems, including nitrogen-fixing bacteria and phosphate-solubilizing bacteria, biochar provides a porous habitat for microbial colonization and enhances microbial stability. At the same time, surface functional groups such as carboxyl groups (-COOH) contribute to interfacial interactions that support microbial activity and nutrient transformation processes in the rhizosphere environment, thereby improving nutrient uptake and soil fertility [67].

Phosphorus transformation processes are also influenced by biochar amendment. Through adsorption and surface complexation, biochar can regulate phosphorus availability in soil, while its interaction with microbial communities further contributes to nutrient cycling and plant growth promotion.

Overall, the remediation of nitrogen and phosphorus pollution by biochar is governed by coupled processes involving microbial enrichment, interfacial electron regulation, and nutrient transformation pathways. Compared with organic pollutants and heavy metals, nutrient cycling systems are more dependent on microbial electron-flow regulation, highlighting the role of biochar as a key mediator in soil biogeochemical processes.

**Table 3:** Summarizes representative biochar-immobilized microorganism/material systems and their reported pollutant-remediation performance.

Biochar/Composite System	Pollutants	Degradation or Removal Efficiency	References
Biomass waste co-pyrolyzed with phosphorus/silicon-containing materials	Cd	Soil-available Cd was reduced by 82%	[49]
Corn biochar loading *Pseudomonas*	Cd/Cu	The immobilization efficiency increased by 35–47% compared with pristine biochar.	[21]
Corn stalk biochar/ g-C_3_N_4_	Cd	Soil-available Cd was reduced by 85%	[50]
Sulfur-modified biochar	Hg^2+^	The adsorption capacity reached 598 mg/g, representing a 3.3-fold increase.	[51]
FeCl_3_-modified Enteromorpha prolifera biochar	Cr(VI)	The adsorption capacity reached 386 mg/g, with a reduction efficiency of 98.2%	[53]
Biochar-assisted modified nanoscale zero-valent iron	Cr(VI)	The removal capacity increased to 412 mg/g.	[54]
FeO-loaded biochar	Cr(VI)	The adsorption capacity reached 498 mg/g, with a reduction efficiency of 99.2%	[68]
Macauba nut shell biochar	U	The adsorption capacity reached 586 mg/g for biochar prepared at 700 °C	[55]
SiBC@γ-Fe_2_O_3_	U(VI)	The adsorption capacity reached 612 mg/g, with a reduction efficiency of 89.5%	[56]
Coconut shell biochar/Fe_3_O_4_-modified biochar/phosphate-modified biochar	Ni	Fe-BC exhibited a Ni adsorption capacity of 72.3 mg/g, which was 1.88 times that of the original BC	[57]
Peanut shell biochar@*Paenarthrobacter* sp. AT5	Atrazine(ATZ)	The degradation efficiency of the combined treatment was 2.8 times higher than that of AT5 alone	[59]
Pine biochar@FBC	BDE-47	The degradation rate increased by 3.2-fold compared with FBC alone	[60]
KOH-activated rape straw biochar@*Aspergillus sydowii* W1	DEHP	A DEHP degradation efficiency of 98.5% was achieved	[61]
SA-BE@BFC	PHE/Cd	The PHE removal efficiency reached 98.5%, and available Cd was reduced by 91%	[64]

## 5. Influencing Factors Affecting Biochar-Mediated Pollutant Remediation

The remediation efficiency of pollutants in biochar-based immobilized systems is controlled by multiple environmental and material-related factors, including biochar physicochemical properties, pyrolysis conditions, solution pH, initial pollutant concentration, and coexisting ions. These factors collectively regulate adsorption behavior, microbial activity, and biochar-mediated interfacial electron-transfer processes. Understanding their roles is essential for optimizing biochar-mediated remediation performance in complex environmental systems. Table 4 summarizes how selected environmental and material-related factors affect the pollutant-remediation performance of biochar-immobilized microorganism/material systems.

### 5.1. Characteristics of Biochar

The physicochemical properties of biochar play a central role in determining its performance as an electron shuttle in pollutant remediation systems. Key properties, including specific surface area, pore structure, surface functional groups, and electrical conductivity, directly influence adsorption capacity and biochar-mediated interfacial electron-transfer efficiency [69,70].

Biochar provides not only physical support for microbial colonization but also an interfacial platform for electron exchange between microorganisms and pollutants. Compared with free microbial systems, biochar-based immobilization systems enhance microbial stability and reduce environmental stress, thereby improving the overall efficiency of pollutant transformation.

These properties are primarily governed by feedstock composition and pyrolysis conditions. Feedstock characteristics (e.g., lignin, cellulose, nutrient, and ash content) determine the formation of carbon structure precursors, while pyrolysis temperature controls the degree of aromatization, graphitization, and functional group retention [71]. As a result, biochars produced under different conditions exhibit distinct electron-shuttling behaviors, ranging from surface functional group-mediated redox processes to conductive carbon matrix-driven electron transport.

#### 5.1.1. Impact of Feedstock Type

Biochars derived from different biomass feedstocks exhibit significant differences in elemental composition, surface chemistry, and electron-shuttling behavior [72]. These differences influence the dominant electron-shuttling pathways in pollutant remediation systems.

Lignocellulosic biomass (e.g., wood) is rich in cellulose, hemicellulose, and lignin. During pyrolysis, cellulose and hemicellulose decompose to form oxygen-containing intermediates with reducing properties, which can participate in initial electron-donation processes. At higher pyrolysis temperatures, lignin is transformed into aromatic carbon structures with extended π-conjugation networks. These graphitized domains enhance electron delocalization and conductivity, thereby promoting long-range electron transport through the biochar matrix.

Herbaceous biomass-derived biochars generally contain higher ash contents and more diverse surface chemistry. Metal species in ash (e.g., K, Ca, Mg) can exist as oxides or salts, which may participate in interfacial electron transfer by acting as catalytic or electron-mediating sites. In addition, heteroatom-containing functional groups (e.g., nitrogen-containing groups) contribute to surface redox activity by acting as both electron donors and acceptors, thereby enhancing surface-mediated electron-transfer processes.

In contrast, manure-derived biochars typically contain higher levels of organic residues and heteroatoms such as nitrogen and sulfur. These components influence electron distribution within the carbon matrix and enhance surface redox reactivity. However, due to their relatively lower structural ordering, such biochars generally exhibit weaker intrinsic conductivity and are therefore more dependent on microbial cooperation in microbially mediated electron-transfer remediation systems.

Overall, the differences in feedstock composition contribute to variations in electron-shuttling mechanisms in biochars, ranging from conductivity-dominated electron transport in wood-derived biochars to functional-group-mediated redox cycling in herbaceous and manure-derived biochars. This is consistent with previous findings showing that high-temperature wood-derived biochars favor electron-transfer-mediated Cr(VI) reduction, whereas low-temperature manure-derived biochars are more effective in microbially mediated immobilization processes [73]. However, inconsistent conclusions regarding the superiority of different feedstock-derived biochars have also been reported. These discrepancies may result from variations in biomass composition, ash content, pyrolysis conditions, and evaluation methods. Therefore, feedstock type alone cannot fully determine electron-shuttling performance, and its effects should be considered together with carbon structure, surface chemistry, and environmental conditions.

#### 5.1.2. Pyrolysis Temperature

Pyrolysis temperature is a key factor governing the physicochemical properties of biochar and plays a decisive role in regulating its electron-shuttling behavior [74]. It primarily controls the balance between surface functional group density and carbon structural ordering, thereby influencing the dominant electron-shuttling mechanism. At low to medium pyrolysis temperatures, biochar retains a high abundance of oxygen-containing functional groups such as hydroxyl, carboxyl, and carbonyl groups. These redox-active moieties facilitate electron exchange through surface-mediated redox cycling processes. In this regime, electron transfer mainly occurs via localized electron-donation and electron-acceptance reactions at the biochar surface, which enhances indirect electron transfer pathways in pollutant transformation.

As the pyrolysis temperature increases, dehydration and decarboxylation reactions progressively reduce surface functional group density, while promoting the formation of condensed aromatic structures. At high temperatures, the carbon matrix becomes increasingly graphitized, forming extended π–electron-conjugation networks. These conductive domains significantly enhance electron delocalization and enable long-range electron transport within the biochar structure [75].

Therefore, pyrolysis temperature induces a shift in electron-shuttling mechanisms from surface functional group-mediated redox cycling at lower temperatures to conductivity-driven electron transport at higher temperatures. This mechanistic shift is consistent with previous findings that high-temperature biochars exhibit enhanced performance in electron-transfer-mediated pollutant reduction processes, whereas low-temperature biochars are more effective in microbially assisted immobilization systems [76]. However, the effect of pyrolysis temperature is not simply linear. Higher temperatures improve electron conductivity by promoting aromatic carbon formation but may reduce redox-active functional groups involved in electron exchange. Therefore, the optimal pyrolysis condition depends on the dominant electron-transfer pathway, with highly graphitized biochars favoring direct electron transfer and functional-group-rich biochars supporting indirect electron transfer processes. The trade-off between enhanced electrical conductivity and decreased surface redox activity at higher pyrolysis temperatures indicates that electron-shuttling performance cannot be optimized by simply increasing carbonization degree. Instead, the balance between conductive carbon domains and redox-active surface sites should be considered according to the dominant electron-transfer pathway involved in pollutant transformation.

Overall, differences in remediation performance among biochar systems are determined by the combined effects of carbon structure, surface chemistry, pollutant accessibility, and interfacial interactions with microorganisms or minerals.

### 5.2. pH

Solution pH is a critical environmental factor that regulates the electron-shuttling efficiency of biochar-based systems by controlling surface charge distribution, pollutant speciation, and the activity of electron donors and acceptors. These combined effects directly influence adsorption behavior and biochar-mediated interfacial electron-transfer processes.

Under acidic conditions, Cr(VI) mainly exists in the forms of HCrO_4_^−^ and Cr_2_O_7_^2−^. In this environment, protonation of oxygen-containing functional groups (e.g., –COOH and –OH) on the biochar surface leads to a positively charged surface, which enhances electrostatic attraction toward negatively charged Cr(VI) oxyanions [77,78]. This process promotes the initial accumulation of pollutants on the biochar surface, thereby facilitating subsequent electron-transfer reactions.

In addition, acidic conditions favor the activation of electron donors in composite systems such as nanoscale zero-valent iron (nZVI)/biochar. The corrosion of nZVI is accelerated under low pH, leading to enhanced electron release, which significantly improves Cr(VI) reduction efficiency in this composite system. In this system, biochar functions as an interfacial electron mediator that receives electrons from nZVI and transfers them to Cr(VI), thereby overcoming direct contact limitations and enhancing interfacial electron utilization efficiency [79].

In contrast, under alkaline conditions, deprotonation of surface functional groups results in a negatively charged biochar surface, which weakens electrostatic attraction toward Cr(VI) oxyanions and reduces adsorption capacity. Meanwhile, the corrosion of nZVI is inhibited, leading to a decrease in electron release. The resulting electrostatic repulsion and reduced electron availability jointly suppress biochar-mediated interfacial electron-transfer efficiency.

Overall, pH regulates electron-transfer-mediated remediation processes by simultaneously controlling surface charge properties, electron donor activity, and interfacial accessibility. These effects collectively determine the performance of biochar-based single and composite systems in pollutant transformation and immobilization.

### 5.3. Coexisting Ions

Soil environments are chemically complex, and the presence of coexisting ions significantly influences the performance of biochar-based pollutant remediation systems by altering pollutant availability and interfacial interactions. These ions regulate adsorption processes, pollutant speciation, and biochar-mediated interfacial electron-transfer processes, thereby affecting overall remediation efficiency [80].

Cations such as Ca^2+^, Mg^2+^, K^+^, and Na^+^ can compete with heavy metal ions for negatively charged adsorption sites on the biochar surface. This competition reduces the availability of cation exchange sites and weakens the electrostatic attraction between biochar and target metal pollutants. As a result, the initial enrichment of heavy metals on the biochar surface is partially inhibited, which indirectly affects subsequent electron-transfer-mediated immobilization processes.

In addition, anions such as PO_4_^3−^, CO_3_^2−^, and SO_4_^2−^ can react with heavy metal ions to form low-solubility precipitates. This process reduces the concentration of free metal ions in solution and promotes their stabilization in solid phases through precipitation and mineral-associated immobilization. Although this mechanism is not directly related to electron transfer, it alters the chemical availability of electron acceptors and therefore influences interfacial redox reactions in biochar-based systems.

Overall, coexisting ions regulate pollutant remediation efficiency through a combination of adsorption-site competition, chemical precipitation, and indirect modulation of biochar-mediated interfacial electron-transfer processes. These effects highlight the importance of the ionic environment in controlling the performance of biochar-mediated electron-shuttling systems in soil remediation.

### 5.4. Initial Concentration of Pollutants

The initial concentration of pollutants is a key factor influencing biochar-mediated remediation efficiency by regulating adsorption-site utilization capacity, interfacial mass transfer, and the balance between electron donors and electron acceptors within the system [77].

At low pollutant concentrations, reactive species such as Cr(VI) are sparsely distributed in solution, allowing sufficient availability of adsorption sites on the biochar surface, including functional groups and pore structures. Under these conditions, pollutants can efficiently migrate to reactive interfaces, where biochar-mediated electron-transfer processes are facilitated by surface functional groups such as hydroxyl and carboxyl groups. In addition, the graphitized carbon structure of high-temperature biochar provides conductive pathways that support long-range electron transport from the biochar matrix to adsorbed electron acceptors, thereby promoting reduction reactions.

At high pollutant concentrations, adsorption sites on the biochar surface become rapidly occupied, leading to increased competition for active functional groups and pore entrances. Under such conditions, continuous electron donation from surface functional groups may result in their depletion, thereby reducing the availability of redox-active electron-shuttling sites. Meanwhile, excessive loading of electron acceptors can limit electron transfer efficiency within the carbon matrix by increasing interfacial transport resistance and reducing electron utilization efficiency.

Overall, pollutant concentration regulates the efficiency of electron-shuttling systems by controlling the balance among adsorption capacity, electron donor availability, and biochar-mediated interfacial electron-transfer kinetics, thereby significantly influencing the performance of biochar-based remediation systems.

### 5.5. Modification Methods

Modification strategies are widely applied to optimize the electron-shuttling performance of biochar by regulating its surface chemistry, electronic structure, and redox-active interfacial sites. These strategies mainly include chemical, physical, and mineral-based modifications, which collectively determine the efficiency of biochar-mediated interfacial electron-transfer in pollutant remediation systems [81].

Chemical modification primarily regulates surface functional groups and redox-active moieties. Acid treatment introduces abundant oxygen-containing functional groups such as –COOH and –OH, which enhance the electron-donating capacity of biochar and promote surface-mediated redox reactions. In addition, acid etching removes amorphous carbon impurities and increases the surface area, thereby shortening electron transfer distances and improving pollutant accessibility. Alkali treatment modifies surface charge characteristics by generating oxygen-containing anionic groups (e.g., –O^−^), enhancing electrostatic interactions with cationic pollutants and facilitating interfacial electron exchange. Oxidative modification using H_2_O_2_ generates carbonyl and carboxyl groups that participate in reversible redox cycling, thereby sustaining continuous electron-shuttling processes, although excessive oxidation may partially disrupt the carbon framework [82].

Physical modification mainly focuses on improving the graphitization degree and structural ordering of biochar. High-temperature alkali or thermal treatments enhance the development of aromatic carbon domains, thereby improving electrical conductivity and promoting long-range electron transport through the carbon matrix. This conductive network plays a critical role in direct electron transport pathways in pollutant reduction systems.

Mineral or oxide-based modification introduces additional redox-active interfaces into the biochar structure. For example, impregnation with manganese oxides (MnO_x_) and iron oxides (FeO_x_) significantly enhances electron transfer capacity through synergistic interactions between metal oxides and the carbon matrix. These oxides can act as electron donors or acceptors depending on their valence states, while biochar provides conductive pathways and stabilizes interfacial electron redistribution. The coupling between metal oxides and biochar leads to optimized electron-cloud distribution and improved interfacial electron transfer efficiency [83].

Overall, modification strategies enable biochar to function as a tunable electron-shuttling platform by simultaneously regulating surface functional chemistry, carbon conductivity, and interfacial redox activity, thereby significantly enhancing its performance in soil pollutant remediation. However, the improvement of electron-shuttling performance through modification depends on the type and degree of modification. For example, oxidation treatments can introduce oxygen-containing functional groups that enhance surface redox reactions, but excessive oxidation may damage the conductive carbon framework and limit electron transport. Conversely, thermal or carbonization-based modifications can increase graphitic structures and electrical conductivity, while reducing functional groups involved in electron exchange. Therefore, selecting appropriate modification strategies requires balancing electron transport capacity and redox-active site density according to the targeted remediation process. The inconsistent performance of modified biochars reported in previous studies further suggests that excessive enhancement of a single property may not necessarily improve overall remediation efficiency. A rational modification strategy should optimize the synergistic interaction among conductivity, redox activity, pollutant accessibility, and microbial compatibility. Nevertheless, the application of modified biochars requires careful consideration of preparation complexity, economic feasibility, and environmental safety. Chemical modification and metal-based functionalization can enhance electron-transfer performance by introducing additional active sites or redox centers; however, these approaches may involve additional reagents, increase production costs, and potentially introduce concerns related to residual chemicals or metal leaching during long-term soil applications. Therefore, future modification strategies should aim to optimize electron-shuttling performance while maintaining structural stability, environmental compatibility, and practical feasibility.

**Table 4:** Summarizes how selected factors affect the pollutant-remediation performance of biochar-immobilized microorganism/material systems.

Biochar/Composite System	Factor	Pollutant	Degradation or Removal Efficiency	Reference
Peanut shell	Pyrolysis temperatures of (300/500/700 °C)	Cr(VI)	The Cr(VI) reduction efficiency of biochar prepared at 500 °C reaches 98%	[1]
BC- nZVI	Soluble starch (SS) regulates the morphology and electron transfer pathway of nZVI	Cr(VI)	The Cr(VI) reduction efficiency increased from 58% to 95%, and the electron utilization efficiency increased 4.2-fold	[74]
Horse manure	Nano-MgO modification	Uranium-containing wastewater	The adsorption capacity reached 1128 mg/g under acidic conditions, and more than 78% of uranium was adsorbed within 5 min	[76]
Pine cone	NaOH modification	Cr(VI)	A Cr(VI) removal efficiency of 78% was achieved within 10 min	[77]
Sludge	nZVI	Cr(VI) and Cr(III)	The Cr(VI) reduction efficiency reached 98.5%, the Cr(III) immobilization efficiency exceeded 99%, and the electron utilization efficiency increased to 4.8 times that of conventional nZVI	[79]
Peanut shell	Pyrolysis temperature of 400–800 °C	Cr(VI)	Biochar prepared at 800 °C reduced 99% of Cr(VI) within 60 min	[78]
Straw	Modification treatment	Heavy metals and tetracycline	Straw-based biochar reduced Cd and Pb by more than 70% and exhibited high tetracycline adsorption capacity. H_2_O_2_-modified biochar showed the highest efficiency for Cr(VI) reduction in manure-derived biochar systems	[80]
Rice husk, wood chips and mixed biochar	Amino, phosphoric acid, FeCl_3_ modification	Coexisting heavy metals (Pb^2+^/Cd^2+^/Cu^2+^/Ni^2+^)	The Cd^2+^ adsorption capacity increased by 389%, and the Pb^2+^ adsorption capacity increased by 172%	[83]

## 6. Conclusions and Future Outlook

This review summarizes recent advances in the application of biochar as an electron shuttle for soil pollutant remediation, with particular emphasis on biochar-immobilized microbial systems and biochar-based composite materials. A conceptual three-pathway electron transfer framework is outlined, including (i) direct electron transfer via graphitized carbon networks, (ii) indirect electron transfer mediated by surface redox-active functional groups, and (iii) synergistic electron transfer at biochar-based microbial or mineral interfaces. This framework provides a useful perspective for understanding electron transport processes in biochar-mediated environmental systems. Current understanding indicates that biochar-mediated electron shuttling is not governed by a single structural parameter, but rather by the synergistic regulation of conductive carbon domains, redox-active functional groups, interfacial interactions, and environmental conditions.

The high remediation efficiency and environmental sustainability of biochar-based systems are mainly associated with their ability to regulate interfacial electron transfer, shorten pollutant transformation pathways, and reduce reliance on chemical reagents. In addition, the wide availability of biomass feedstocks and the valorization of agricultural waste further support the potential application of biochar within circular economy strategies. The electron-shuttling performance of biochar is strongly influenced by its intrinsic properties (e.g., feedstock type and pyrolysis temperature) and environmental conditions (e.g., pH, coexisting ions, and pollutant concentration), which together affect its redox activity and interfacial reactivity.

Despite extensive progress, quantitative comparison among different biochar-based electron-shuttling systems remains challenging because standardized evaluation criteria for electron-shuttling capability are still lacking. Differences in electrochemical characterization methods, redox-capacity measurements, and experimental conditions may lead to inconsistent interpretations of electron-transfer performance among different studies. More importantly, several critical knowledge gaps remain and should be addressed according to a clear order of research priority:

(1) Unresolved interfacial electron transfer mechanisms: The relative contributions of direct microbial contact and biochar-mediated electron shuttling are still not fully clarified. Advanced techniques, such as in situ electrochemical characterization combined with molecular biology approaches (e.g., knockout of gene encoding key electron transfer proteins), are required to better resolve electron transport pathways at the micro-interface level.

(2) Complex environmental regulation effects: In real soil systems, factors such as pH fluctuations, coexisting ions, and dissolved organic matter as well as mineral interactions and microbial community changes may influence electron transfer processes by altering surface charge distribution or blocking active sites. Long-term evaluation under realistic soil conditions is therefore necessary to determine the environmental adaptability and stability of biochar-based electron transfer systems. Developing structurally stable biochar materials (e.g., via mineral coating or co-pyrolysis strategies) is a promising direction.

(3) Multi-contaminant system complexity: The mechanisms governing the simultaneous removal of heavy metals and organic pollutants remain insufficiently understood. Future studies should focus on designing multifunctional systems that integrate adsorption, redox transformation, and microbial degradation, such as biochar-metal oxide-fungal composites.

(4) Molecular-level regulatory mechanisms: The regulation of microbial electron transfer pathways in biochar systems is still not fully understood. Integrating metagenomics, transcriptomics, and metabolomics may help reveal the expression patterns of key electron transfer genes (e.g., those encoding cytochromes) and their response to biochar interfaces.

(5) Regeneration and life-cycle sustainability: The regeneration behavior of biochar in long-term applications remains unclear. Future research should investigate adsorption–desorption reversibility, electron transfer stability, and structural evolution under repeated use as well as field-scale performance under complex environmental conditions to evaluate the long-term performance of biochar-based remediation systems.

Beyond these knowledge gaps, future studies should establish quantitative relationships between biochar structural characteristics and electron-shuttling performance under realistic environmental conditions. Since electron-transfer behavior is simultaneously affected by carbon structures, surface functional groups, redox-active sites, and environmental factors, distinguishing electron-transfer contributions from adsorption, complexation, precipitation, and microbial effects remains challenging. Therefore, integrated evaluation combining electrochemical measurements, redox-capacity analysis, and pollutant transformation characterization is essential for elucidating biochar-mediated electron-shuttling mechanisms.

Among these unresolved issues, particular emphasis should be placed on establishing standardized and quantitatively comparable evaluation protocols and obtaining direct experimental evidence that distinguishes electron-transfer processes from other simultaneous remediation mechanisms. Building on this foundation, future studies should further clarify quantitative structure-electron-shuttling-remediation relationships and assess their robustness under realistic soil conditions. Greater attention is also needed to molecular-level microbial regulation, multi-contaminant interactions, and long-term field performance, including regeneration capacity, environmental safety, economic feasibility, and life-cycle sustainability.

Overall, biochar-mediated electron transfer provides a framework for understanding the interactions among biochar properties, electron transfer pathways, and pollutant transformation processes. Further research should connect microscale electron transfer mechanisms with environmental-scale remediation performance to support the development of biochar-based remediation strategies. Emerging approaches, including operando characterization, multiscale modeling, and data-driven strategies, may further improve the understanding, prediction, and optimization of biochar-based electron-transfer systems by enabling rational material design and mechanism-guided environmental applications. Future studies should prioritize the establishment of standardized electrochemical evaluation protocols, long-term validation under realistic soil conditions, and the integration of computational and data-driven approaches to predict biochar properties and optimize electron-shuttling performance for specific remediation applications. Together, these approaches can support a progressive research framework encompassing standardized measurement, mechanistic verification, validation under realistic environmental conditions, and field-scale assessment. Furthermore, the practical implementation of biochar-based electron-transfer systems requires consideration of economic feasibility and environmental safety. The cost associated with biomass pretreatment, pyrolysis processes, and functional modification may influence large-scale application. In addition, potential environmental risks related to the release of residual inorganic components or engineered functional materials should be systematically evaluated during long-term use. Integrating life-cycle assessment, cost analysis, and field-scale validation will be essential for promoting sustainable biochar-based remediation technologies.

## Figures and Tables

**Table 1:** Comparison of recent reviews on biochar-based environmental remediation and the perspective of this review.

Reference	Review Focus	Main Contribution	Difference from This Review
[9]	Biochar for soil remediation	Summarized pollutant removal and environmental applications	Does not establish a systematic electron-shuttling framework
[4]	Biochar-based contaminant removal	Focused on adsorption and immobilization mechanisms	Limited discussion of electron-transfer-driven transformation
[10]	Biochar-microbe interactions	Reviewed microbial enhancement and remediation effects	Interfacial electron transfer was not systematically discussed
[11]	Biochar intrinsic properties and electron transfer	Discussed structural factors controlling electron transfer behavior	Did not comprehensively connect electron transfer properties with diverse pollutant remediation processes
This review	Biochar as an electron shuttle	Integrates structure, electron transfer, and remediation mechanisms	Establishes a three-pathway electron transfer framework including direct, indirect, and composite interfacial electron transfer

**Table 2:** Biochar-based systems for pollutant remediation and their dominant mechanisms.

Raw Material	Microorganism/Material	Pollutant	Degradation or Removal Efficiency	Reference
N-doped lignin biochar	None	Cr(VI)	Adsorption coupled with redox transformation promoted Cr(VI) removal through N-containing active sites; 99.9% removal was achieved for 50 mg/L Cr(VI)	[15]
Corn straw	Denitrifying bacteria	Nitrite	Biochar-supported microbial systems enhanced nitrogen transformation and microbial electron utilization; N_2_O reductase gene expression increased 3.2-fold	[14]
Rice husk	Immobilized Alcaligenes faecalis JH1	Phenol	Biochar immobilization enhanced microbial degradation and pollutant accessibility; degradation rate reached 42 mg/(L·h)	[12]
Corn straw	Geobacter sulfurreducens PCA	Pentachlorophenol	Biochar-mediated extracellular electron transfer enhanced microbial dechlorination; PCP dechlorination rate increased 8.4-fold	[23]
nan	Rice husk	Trichloroethylene	Biochar improved microbial electron utilization efficiency during TCE transformation, increasing electron utilization from <40% to >89%	[24]
Pig manure	Thermophilic and alkali-tolerant strain Bacillus halotolerans ACY	Phenol	Biochar-supported microbial degradation improved phenol removal, achieving 99.5% efficiency through enhanced microbial colonization	[25]
Grapefruit peel	Acid-tolerant Acinetobacter	Fe^2+^, Mn^2+^ and NH_4_^+^	Biochar immobilization coupled with microbial transformation enhanced removal efficiencies (>99%, >98%, and >95%, respectively)	[26]
Corn straw	Sulfurized nano-zero-valent iron	Tetrabromobisphenol A	Electron-transfer-assisted reduction coupled with adsorption achieved >99% degradation within 30 min	[28]
Rice straw	Zero-valent iron	Cr(VI)	Fe-mediated electron transfer promoted Cr(VI) reduction to Cr(III); more than 99% of Cr(VI) was reduced within 30 min	[35]
Rice husk	Pyrite	Cr(VI)	Fe/S-mediated redox reactions and precipitation contributed to Cr(VI) immobilization (>99% removal)	[31]
Eucalyptus sawdust	Fe_3_O_4_	Gas-phase p-xylene	Biochar-supported Fe_3_O_4_ enhanced catalytic degradation through interfacial interactions; degradation efficiency reached 96%	[32]
Pomelo peel	TiO_2_	Typical dyes, antibiotics	Biochar-supported photocatalytic systems improved charge separation and degradation efficiency (>98.8%)	[33]
Spartina alterniflora	ZnO/TiO_2_	Antibiotics	Biochar-supported semiconductor composites enhanced photocatalytic degradation (>99.2%) through improved charge transfer	[34]

## Data Availability

The raw data supporting the conclusions of this article will be made available by the authors on request.
